# Clinicopathologic characteristics and long-term prognosis of intraductal papillary neoplasm of the bile duct: a retrospective study

**DOI:** 10.1186/s40001-023-01102-w

**Published:** 2023-03-21

**Authors:** Xin Wu, Binglu Li, Chaoji Zheng

**Affiliations:** Department of General Surgery, Peking Union Medical College Hospital, Chinese Academy of Medical Sciences and Peking Union Medical College, No. 1 Shuaifuyuan, Dongcheng District, Beijing, 100730 China

**Keywords:** Intraductal papillary neoplasm of the bile duct, Intrahepatic, Extrahepatic, Cumulative survival rate, Prognosis

## Abstract

**Background:**

Intraductal papillary neoplasm of the bile duct (IPNB) is a premalignant neoplasm that can involve both the intrahepatic and extrahepatic bile ducts. Owing to the low incidence and confusing nomenclature, its clinicopathological features remain controversial. Additionally, only a few studies have reported on the long-term prognosis of IPNB to date. Therefore, the present study aimed to clarify the clinicopathological characteristics and prognosis of IPNB.

**Methods:**

Medical records of patients with IPNB treated at our hospital between August 2000 and October 2021 were retrospectively reviewed. A database of demographic characteristics, test results, surgical details, pathological findings, and follow-up information was constructed for analysis. Patients were divided into intrahepatic and extrahepatic groups, and dysplasia and invasive carcinoma groups for comparison. Differences between study groups were analyzed using the χ^2^ test, Fisher’s exact test, t-test, or Mann–Whitney U test, as appropriate. Cumulative survival rates were estimated using the Kaplan–Meier method.

**Results:**

In total, 43 patients (21 men and 22 women) with IPNB were included in the study. The median age at diagnosis was 62 (54–69) years. Thirty-eight patients underwent surgery. The mean operation time was (269.5 ± 94.9) min. Five patients underwent endoscopic retrograde cholangiopancreatography for biopsy. Twenty-one and 22 patients had intrahepatic and extrahepatic lesions, respectively. The extrahepatic group had more patients with intraluminal masses (*p* = 0.021) and abnormal bilirubin levels (*p* = 0.001), but fewer patients with hepatolithiasis (*p* = 0.021). The operation time was longer in patients with extrahepatic lesions (*p* = 0.002). Twenty patients had dysplasia and 23 had invasive carcinoma. The invasive carcinoma group had a longer operation time than the dysplasia group (*p* = 0.004). As of March 2022, 39 patients were followed up, with a mean follow-up time of (56.2 ± 38.2) months. Fifteen patients survived without tumors, two survived with tumors, and 22 patients died. The 1-, 3-, 5-, and 10-year cumulative overall survival rates were 86.9%, 65.8%, 49.8%, and 32.0%, respectively.

**Conclusions:**

IPNB is a rare bile duct disease that occurs mainly in patients with advanced age. Surgery is the primary treatment strategy. Intrahepatic and extrahepatic lesions, as well as dysplasia and invasive carcinoma have their own unique characteristics. The long-term prognosis of IPNB is generally poor.

## Background

Intraductal papillary neoplasm of the bile duct (IPNB) is an independent pathological entity that can involve both extrahepatic and intrahepatic bile ducts [1, 2]. It is characterized by intraductal papillary or villous growth of biliary epithelium with fine fibrovascular cores [1, 3]. The tumor may be asymptomatic or cause symptoms by blocking the bile duct lumen. Before being officially named in 2010, IPNB was known by a number of other names, including biliary papilloma if the tumor was solitary and biliary papillomatosis if the tumors were multiple [4]. According to the World Health Organization 2019 Classification, IPNB is a premalignant neoplasm, and the lesion with invasive features is defined as IPNB with associated invasive carcinoma [1, 2, 5]. IPNB accounts for only 4–38% of all bile duct tumors [6]. Based on the similarities between IPNB, intraductal papillary mucinous neoplasm of the pancreas, and extrahepatic papillary bile duct cancer, two subclassifications of IPNB were proposed by the Japan–Korea IPNB study group in 2018 [7]. Both IPNB and other subtypes of cholangiocarcinoma are geographically prevalent in the Eastern countries [6, 8, 9], though the former has a better prognosis than the latter [4].

Since it was first proposed by Zen et al. in 2006 [10], an increasing number of studies have focused on IPNB. However, due to the low incidence and confusing naming methods, the clinical presentation, diagnostic approach, histopathologic characteristics, and long-term prognosis of IPNB are still controversial [1, 11]. Thus, the present study aimed to analyze the clinicopathological features and prognosis of IPNB to provide a deeper understanding of this rare disease.

## Methods

This study only involves the collection of existing data. The information is recorded by the investigator in such a manner that subjects cannot be identified, directly or through identifiers linked to the subjects. There is no intervention. It was approved by the institutional review board of Peking Union Medical College Hospital (I-22PJ117). The requirement for informed consent for publication of patient data was waived due to the retrospective nature of the study.

### Patients

We reviewed the medical records of patients with bile duct tumors treated at our hospital between August 2000 and October 2021 for this single-center retrospective observational study. Patients were selected based on the following inclusion criteria: (1) extrahepatic or intrahepatic bile duct tumor diagnosed by preoperative imaging examination, (2) radical surgery or biopsy performed at our hospital, (3) IPNB confirmed via postoperative pathology, and (4) availability of complete medical records. Patients with coexisting digestive system tumors were excluded from the study. Clinical data were compiled from outpatient and inpatient medical records. A retrospective database containing demographic characteristics, clinical presentation, laboratory and imaging test results, operative details, pathological results, and follow-up information was constructed for the analysis.

### Treatment and follow-up

Preoperative liver function parameters, serum bilirubin levels, and at least one computerized tomography (CT) and magnetic resonance imaging (MRI) scan were performed for every patient. The treatment strategy was determined based on imaging test results, patients’ general health condition, and willingness to undergo treatment. For patients who underwent surgery, the surgery was performed under general anesthesia in the supine position. Hepatic lobectomy and hemihepatectomy were performed in patients with intrahepatic lesions according to the tumor location. Pancreaticoduodenectomy and radical choledochal resection combined with hepaticojejunostomy were performed in patients with extrahepatic lesions based on the location of the tumor in the common bile duct. An intraoperative frozen section was obtained to ensure negative margins. Endoscopic retrograde cholangiopancreatography was performed for retrieving biopsy specimens from patients who did not undergo surgery. Paraffin embedded sections of the surgical specimens were examined by two independent pathologists before the final pathological diagnoses were confirmed and disputed data were resolved by discussion. Patients were followed up every 3 months for the first year after surgery and then every 6 months thereafter via outpatient interviews and telephone calls. Blood tests, ultrasonography, CT, and MRI were used for follow up examinations.

### Definition

According to the 5th edition of World Health Organization Classification, IPNB was defined as a grossly visible premalignant neoplasm with intraductal papillary or villous growth of biliary-type epithelium and histologically thin fibrovascular stem [1, 5]. According to the pathological results, all patients with IPNB were classified into three grades: low-intermediate dysplasia, high-grade dysplasia, and IPNB with associated invasive carcinoma [5]. The first two were defined as dysplasia, and the last as invasive carcinoma. The Cancer Staging Manual (8th edition) of the American Joint Committee on Cancer was used to assign tumor stage [12, 13]. Complications after surgery were defined as any deviation from the normal postoperative course and classified according to the Clavien-Dindo system [14]. The reference levels of alanine transaminase, gamma-glutamyl transpeptidase, alkaline phosphatase, total bilirubin, direct bilirubin, carcinoembryonic antigen (CEA), and carbohydrate antigen 19–9 (CA19-9) were ≤ 40U/L, ≤ 45U/L, ≤ 100U/L, ≤ 22.2 µmol/L, ≤ 6.8 µmol/L, ≤ 5 µg/L, and ≤ 34U/mL, respectively. Tumor metastases and loco-regional recurrence were diagnosed by imaging tests, and pathological evidence was not required.

### Statistical analysis

All statistical analyses were performed using Statistical Package for Social Sciences software (version 25.0; IBM Corp., Armonk, NY, USA). Continuous variables with normal and skewed distributions were shown as mean ± standard deviation and median (25^th^–75^th^ percentiles), respectively. Categorical variables were described as absolute numbers and frequencies. Differences between study groups were analyzed using the χ^2^ test, Fisher’s exact test, Student’s t-test, or Mann–Whitney U test, as appropriate. Survival probability was estimated using the Kaplan–Meier method. Statistical significance was set at *p* < 0.05.

## Results

A total of 43 patients with IPNB treated at our hospital between August 2000 and October 2021 were selected for the final analysis based on the inclusion and exclusion criteria. Among these, 21 were men (48.8%) and 22 were women (51.2%) with a male-to-female ratio of 1:1.05. The median age at diagnosis was 62 (54–69) years. Thirty-four (79.1%) patients presented with preoperative symptoms. Jaundice was the most common symptom, occurring in 26 patients (60.5%), followed by abdominal pain in 18 patients (41.9%), and fever in 17 patients (39.5%). Nine patients (20.9%) were asymptomatic and were diagnosed during routine medical examination. Six (14.0%) and five (11.6%) patients had coexisting choledocholithiasis and hepatolithiasis, respectively. Three (7.0%) patients had pancreaticobiliary maljunction. None of the patients had a medical history of clonorchiasis or worked in the printing industry. Abnormal preoperative liver function test results and serum bilirubin levels were observed in 24 (55.8%) and 19 (44.2%) patients, respectively. CEA levels were elevated in 10 (23.3%) patients and CA19-9 levels were elevated in 26 (60.5%) patients.

Thirty-eight patients underwent surgery. Hepatic lobectomy and hemihepatectomy were performed in 20 patients, pancreaticoduodenectomy in 11 patients, and radical choledochal resection combined with hepaticojejunostomy in 7 patients. The mean operation time was (269.5 ± 94.9) min (range: 85–480 min), and the median intraoperative bleeding was 300 (162.5–775) mL (range: 50–2000 mL). Postoperative complications occurred in eight patients, including gastroparesis (n = 2), intra-abdominal bleeding (n = 2), fever (n = 1), pleural effusion (n = 1), bile duct infection (n = 1), and wound infection (n = 1). Of these, one patient with intra-abdominal bleeding underwent reoperation, while the others were treated conservatively. According to the Clavien-Dindo classification, three patients were classified as grade I, four as grade II, and one patient was classified as grade IIIb. Five patients refused surgery and endoscopic retrograde cholangiopancreatography was used to perform biopsy in these patients. All 43 patients were pathologically diagnosed as having IPNB. Twenty-one (48.8%) and 22 (51.2%) patients had intrahepatic and extrahepatic lesions, respectively. Twenty (46.5%) patients had dysplasia (low-intermediate dysplasia, n = 15; high-grade dysplasia, n = 5), while 23 (53.5%) had invasive carcinoma. The resection margin was positive in 5 patients. Lymph node dissection was performed in 24 patients, and a total of 215 lymph nodes were removed, including three positive nodes. Eighteen patients had definite mucous secretion, while the others had no mucous secretion or related data were not available.

**Fig. 1:**
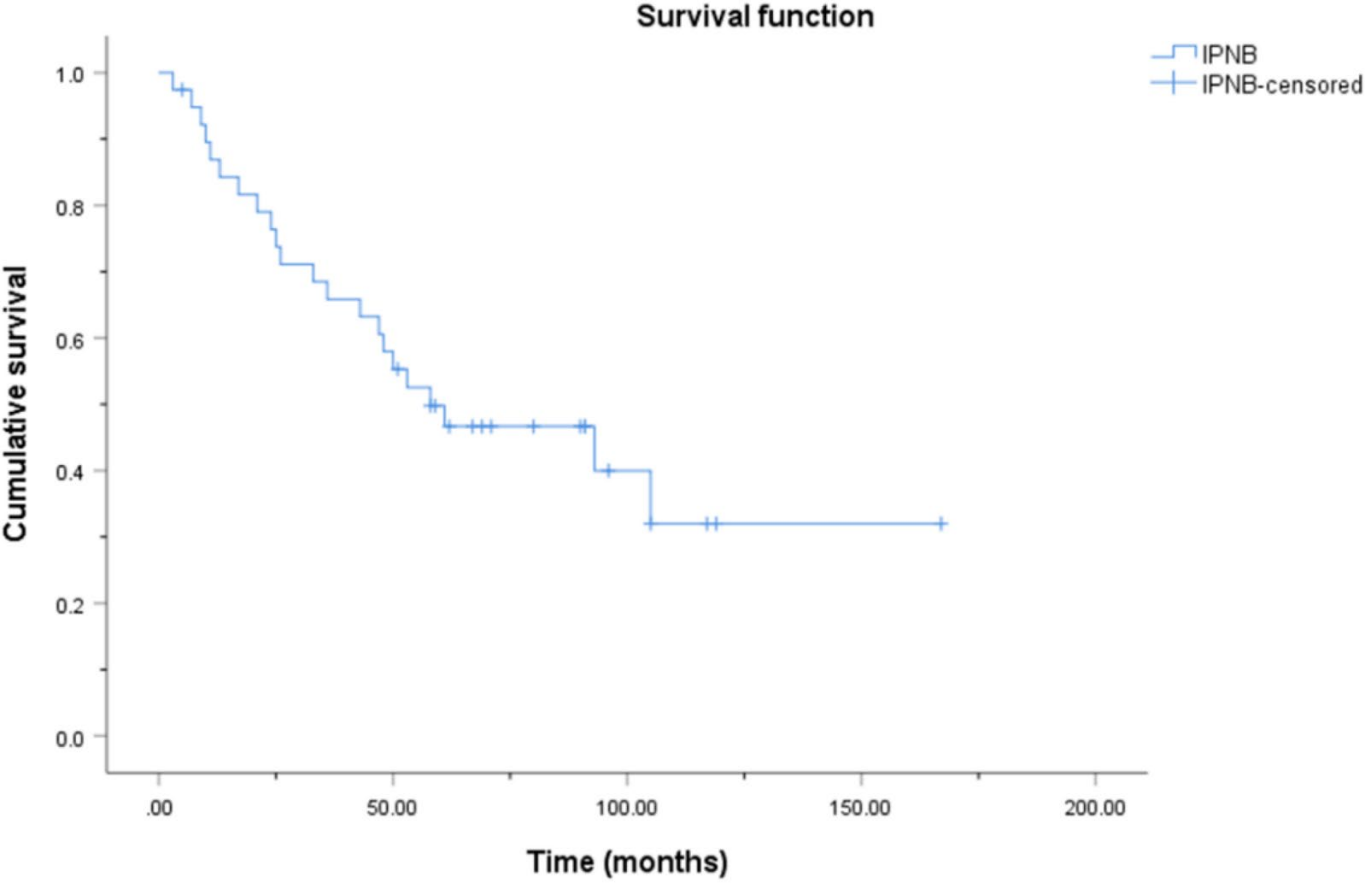
Kaplan–Meier overall survival curve for patients with IPNB. The 1-, 3-, 5-, and 10-year cumulative overall survival rates were 86.9%, 65.8%, 49.8%, and 32.0%, respectively

As of March 2022, 39 patients were followed up with a mean follow-up time of (56.2 ± 38.2) months (range: 3–167 months). Fifteen patients (38.5%) survived without tumors, two patients (5.1%) survived with tumors, 22 patients (56.4%) died and four patients were lost to follow-up. For the 39 patients who were followed up, the cumulative overall survival (OS) rates are demonstrated through a Kaplan–Meier curve (Fig. 1). The 1-, 3-, 5-, and 10-year cumulative OS rates were 86.9%, 65.8%, 49.8%, and 32.0%, respectively. The estimated median survival time was 58.0 months. Of the 22 patients who died, nine patients with invasive carcinoma died of tumor metastases, four patients with invasive carcinoma died of tumor metastases and loco-regional recurrence, one patient with high-grade dysplasia died of loco-regional recurrence, five patients with dysplasia died of biliary obstruction and infection, and the exact cause of death for the remaining three patients with dysplasia was unknown. It is worth mentioning that all five patients with positive resection margins (invasive carcinoma, n = 4; high-grade dysplasia, n = 1) had loco-regional recurrence and died, and one patient with lymph nodes metastases died. For patients with biliary obstruction, percutaneous transhepatic cholangial drainage was used to relieve symptoms.

Based on the lesion location, patients were divided into intrahepatic (n = 21) and extrahepatic (n = 22) groups. The comparison of clinicopathological features of patients in the two groups is shown in Table 1. The extrahepatic group had more patients with intraluminal masses (*p* = 0.021) and abnormal bilirubin levels (*p* = 0.001), but fewer patients with hepatolithiasis (*p* = 0.021). The operation time was longer in patients with extrahepatic lesions (*p* = 0.002). Based on the tumor nature, patients were divided into dysplasia (n = 20) and invasive carcinoma (n = 23) groups, and the two groups are compared in Table 2. The invasive carcinoma group had a longer operation time than the dysplasia group (p = 0.004).

**Table 1:** Comparison of IPNB patients with intrahepatic and extrahepatic lesions

	Lesion location		P
	Intrahepatic (n = 21)	Extrahepatic (n = 22)	
Male/female (n)	10/11	11/11	0.876
Age (years)	62 (59–68)	63 (50.3–71.3)	0.884
BMI (kg/m^2^)	23.8 ± 3.4	24.9 ± 3.5	0.312
Symptoms (n)			
Abdominal pain	10	8	0.455
Jaundice	11	15	0.289
Fever	11	6	0.092
Imaging features (n)			
Choledocholithiasis	4	2	0.412
Hepatolithiasis	5	0	0.021
Biliary dilatation	21	19	0.233
Intraluminal mass	6	14	0.021
Biliary stricture	4	5	1.000
Biliary wall thickening	2	7	0.132
Abnormal liver function (n)	9	15	0.095
Abnormal bilirubin (n)	4	15	0.001
CEA > 5 µg/L (n)	5	5	1.000
CA19-9 > 34 U/mL (n)	10	16	0.092
Operation time (min)	226.5 ± 75.3	317.2 ± 93.2	0.002
Patients without surgery (n)	1	4	0.345
Dysplasia / invasive carcinoma (n)	10/11	10/12	0.887
Positive margin (n)	3	2	0.664
Survival (n)	8	9	0.850
Survival without tumor (n)	7	8	0.835

*IPNB* intraductal papillary neoplasm of the bile duct, *BMI* body mass index, *CEA* carcino-embryonic antigen, *CA19-9* carbohydrate antigen 19–9

**Table 2:** Comparison of IPNB patients with dysplasia and invasive carcinoma

	Dysplasia (n = 20)	Invasive carcinoma (n = 23)	P
Male/female (n)	10/10	11/12	0.887
Age (years)	60 (50.5–72)	64 (55–68.5)	0.609
BMI (kg/m^2^)	23.9 ± 3.3	24.7 ± 3.7	0.438
Symptoms (n)			
Abdominal pain	8	10	0.818
Jaundice	10	16	0.191
Fever	7	10	0.571
Imaging features (n)			
Choledocholithiasis	3	3	1.000
Hepatolithiasis	3	2	0.650
Biliary dilatation	18	22	0.590
Intraluminal mass	11	9	0.298
Biliary stricture	6	3	0.263
Biliary wall thickening	5	4	0.711
Abnormal liver function (n)	8	16	0.052
Abnormal bilirubin (n)	9	10	0.920
CEA > 5 µg/L (n)	3	7	0.405
CA19-9 > 34 U/mL (n)	11	15	0.494
Operation time (min)	219.7 ± 77.5	305.7 ± 91.2	0.004
Patients without surgery (n)	4	1	0.167
Intrahepatic/extrahepatic (n)	10/10	11/12	0.887
Positive margin (n)	1	4	0.351
Survival (n)	10	7	0.191
Survival without tumor (n)	10	5	0.052

*IPNB* intraductal papillary neoplasm of the bile duct, *BMI* body mass index, *CEA* carcino-embryonic antigen, *CA19-9* carbohydrate antigen 19–9

## Discussion

The present study revealed that IPNB mainly involved patients with advanced age, and there was no difference in occurrence between men and women. The most common preoperative symptom was jaundice in our study group. The majority of patients were treated with surgery and lymphatic metastasis was very rare. Compared with intrahepatic group, extrahepatic group had more patients with intraluminal masses, abnormal bilirubin levels, and longer operation time, but fewer patients with hepatolithiasis. Compared to the dysplasia group, the invasive carcinoma group had a longer operation time. The 1-, 3-, 5-, and 10-year cumulative OS rates for IPNB were 86.9%, 65.8%, 49.8%, and 32.0%, respectively.

IPNB is a very rare variant of bile duct tumors with a high incidence in Asian populations [3]. It is very difficult to clarify the precise clinical features and prognosis of IPNB owing to its low incidence. In a multicenter study, Kubota et al. [3] summarized clinical features of IPNB in 694 patients and found that the median age of the patients was 67 years with a male-to-female ratio of 1.74:1. In another multicenter study which included 387 patients, the mean age was 65.5 ± 8.4 years and the male to female ratio was of 1.73:1 [6]. The present study revealed a similar age of disease onset, but the proportion of male involvement was lower. The clinical presentation of IPNB is nonspecific and mainly depends on the tumor location. Common symptoms include abdominal pain, fever, and jaundice [15]. The preoperative diagnosis of IPNB chiefly depends on imaging examination, and the typical imaging findings are biliary dilatation and an intraluminal mass. CT and MRI are commonly used imaging methods, and the latter has significant advantages in terms of differentiating benign lesions from malignant lesions [16, 17]. In the present study, the clinical presentation and imaging findings of IPNB were consistent with those reported previously. Endoscopic ultrasound is another important imaging modality used in the diagnosis of IPNB, as it can show the depth of tumor invasion and guide mucus aspiration [11, 18]. In a previous study, spyglass and endoscopic endoluminal radiofrequency ablation were combined to increase early diagnosis rates and provide more therapeutic options. This combination was found to be effective in selected patients [19]. Despite all the research that has been done till date, the etiology of IPNB is still unclear. Certain factors, such as choledocholithiasis, hepatolithiasis, and clonorchiasis were previously thought to be associated with IPNB, but none of them presented significant association in our study. Youn et al. [20] studied 146 patients with intrahepatic IPNB and found that only 18 patients (12.3%) had hepatolithiasis. Further studies are required to determine the etiology of IPNB. At present, surgery is the recommended treatment for IPNB. In a meta-analysis of 57 studies with 476 patients, up to 43% of the patient had IPNB with associated invasive carcinoma [11]. In our study, 53.5% (23/43) of the patients had invasive carcinoma. Early surgery is advisable for radiologically suspected IPNB to achieve radical excision and satisfactory outcome.

IPNB with different lesion locations (intrahepatic and extrahepatic) and tumor nature (dysplasia and invasive carcinoma) have different characteristics. Extrahepatic lesions are more likely to express jaundice and abnormally elevated CA19-9 levels, and malignant lesions are more likely to have jaundice with abnormally elevated levels of both CEA and CA19-9 [6]. You et al. [21] retrospectively reviewed 103 patients with IPNB and found that extrahepatic lesions were more commonly associated with an invasive disease and a worse prognosis than intrahepatic lesions. It is known that the prognosis of IPNB is better than that of cholangiocarcinoma. Harada et al. [22] studied 22 patients with IPNB and 391 patients with cholangiocarcinoma, and reported that the 10-year survival rates of benign IPNB, malignant IPNB, and cholangiocarcinoma were 100%, 69%, and 38%, respectively. Kim et al. [6] reported that the cumulative 1-, 3-, and 5-year OS rates for IPNB were 97.2%, 89.6%, and 80.9%, respectively. Luvira et al. [23] retrospectively analyzed 148 patients with IPNB and found that the 1-, 3-, and 5-year OS rates were 83.6%, 64.4%, and 47.0%, respectively. Krawczyk et al. [2] performed a comprehensive review of IPNB and reported a 5-year survival rate of 53.6%. The long-term prognosis of IPNB in this study was revealed to be worse than that reported by Kim et al. but similar to that reported by Luvira et al. and Krawczyk et al. This might be related to the difference in the number of patients with malignant lesions in the different studies. In the present study, invasive carcinoma and high-grade dysplasia accounted for the majority of patients, which was an important reason for the high mortality. It has been suggested that the prognosis of IPNB may be associated with tumor invasiveness, lymph node metastasis and bile duct margin status [1, 6]. Lymph node metastasis was uncommon in IPNB, and it was an independent prognostic factor [1]. Bile duct margin was also associated with prognosis in patients with IPNB. Even ductal margin with carcinoma in situ had higher tumor recurrence rate than negative margin [1]. Intraductal dissemination is also known to be an important factor in the recurrence and prognosis of IPNB [24]. Univariate and multivariate analyses of prognostic factors were not performed in the present study due to the limited number of patients.

In the present study, among dead patients without tumor metastases, one died of loco-regional recurrence and five died of biliary obstruction and infection. Because loco-regional recurrence was diagnosed by imaging tests and pathological evidence was not required, there might be bias on diagnosing of death causes. The exact cause of biliary obstruction could be controversial in some patients, and it was difficult to distinguish benign stenosis and loco-regional recurrence. In previous studies, benign stenosis was reported to be rare [25, 26]. The five patients who died of biliary obstruction and infection might also have loco-regional recurrence. The recurrence or existence of IPNB after primal surgery in this study might be underestimated. In the treatment of patients with biliary obstruction, whatever the cause, the first thing to do is to establish proper bile flow. According to the general condition of the patients, the resectability of local lesions, and whether there is distant metastasis, a comprehensive judgment should be made to select the appropriate treatment such as reoperation and percutaneous transhepatic cholangial drainage.

IPNB is diagnosed and classified according to the specific pathological findings of the lesion. The traditional classification of IPNB is similar to that of intraductal papillary mucinous neoplasms of the pancreas. According to microscopic morphology and different biomarkers, IPNB can be divided into gastric, oncocytic, pancreatobiliary, and intestinal types [27]. More than one pathological type can be observed in a single IPNB specimen, and some cases might contain all the four types [28]. To further clarify the characteristics of different IPNB types and their relationship with disease prognosis, experts from Japan and Korea have developed a new classification system with two subtypes [7]. Type 1 commonly develops in the intrahepatic bile ducts and is histologically similar to intraductal papillary mucinous neoplasm of the pancreas, while type 2 commonly involves the extrahepatic bile ducts and has a more complex histological architecture. IPNB associated with invasive cancer is more common in the type 2 lesions. Most previous studies found that type 1 neoplasm had a better prognosis than type 2 [3, 29–31]. However, it is difficult to differentiate type 1 neoplasms from type 2 neoplasms [31]. In the present study, patients with IPNB were not classified or compared on the basis of the different subtypes, as we did not have sufficient information regarding tumor classification for some patients due to the long study time span.

The present study had some limitations. First, the sample size and investigated variables could not be planned beforehand owing to the retrospective nature of the study. Second, the patient volume was limited because of the low incidence of IPNB and the single-center study design. Third, details pertaining to pathological classification of the neoplasm were insufficient in some cases. Multicenter studies should be performed in the future to obtain more reliable and robust data.

## Conclusions

IPNB is a rare bile duct disease that occurs mainly in patients with advanced age. Jaundice is the most common preoperative symptom and surgery is the primary treatment strategy. Extrahepatic lesions are more likely to show intraluminal masses, and abnormal bilirubin levels than intrahepatic lesions; however, hepatolithiasis is less commonly seen in patients with extrahepatic lesions. The 1-, 3-, 5-, and 10-year cumulative OS rates for IPNB were 86.9%, 65.8%, 49.8%, and 32.0%, respectively. The long-term prognosis is generally poor.

## Data Availability

All data generated or analyzed during this study are included in this published article.

## Abbreviations

IPNB: Intraductal papillary neoplasm of the bile duct
CT: Computerized tomography
MRI: Magnetic resonance imaging
CEA: Carcino-embryonic antigen
CA19-9: Carbohydrate antigen 19–9
OS: Overall survival
